# *Trichinella spiralis* excretory-secretory proteins induced autophagy via activating AMPK/mTOR pathway and protected gut epithelial barrier

**DOI:** 10.1371/journal.pntd.0013863

**Published:** 2025-12-22

**Authors:** Xin Zhuo Zhang, Yao Zhang, Ru Zhang, Jin Yi Wu, Pei Kun Cong, Shao Rong Long, Ruo Dan Liu, Zhong Quan Wang, Jing Cui

**Affiliations:** Department of Parasitology, School of Basic Medical Sciences, Zhengzhou University, Zhengzhou, China; University of Liverpool, UNITED KINGDOM OF GREAT BRITAIN AND NORTHERN IRELAND

## Abstract

**Background:**

*Trichinella spiralis* is an intestine- and tissue-dwelled parasitic nematode, the adult worms (AW) and muscle larvae parasitize in intracellular niche of intestinal epithelium and skeletal muscles of the same host, respectively. Intestinal infective larvae (IIL) and AW are two important enteral stages in *T. spiralis* infection. Their excretory-secretory proteins (ESP) disrupted host’s intestinal epithelial barrier and mediated worm invasion. Meanwhile, *T. spiralis* could induce autophagy of murine intestinal epithelial cells. Autophagy usually plays a role in maintaining the structural and functional integrity of intestinal epithelial barrier. However, the function of autophagy in *T. spiralis* invasion and colonization in host remains unclear. The aim of this study was to investigate whether *T. spiralis* ESP induce enterocyte autophagy and whether ESP-induced autophagy protects intestinal epithelial barrier from ESP-induced destruction.

**Methodology/principal findings:**

The results of qPCR, Western blot and intracellular Ca^2+^ concentration assay showed that IIL and AW ESP induced autophagy of Caco-2 and RAW264.7 cells via increasing RACK1 expression and intracellular Ca^2+^ concentration, and activating AMPK/mTOR pathway. The results of qPCR, Western blot, indirect immunofluorescence test (IIFT), trans-epithelial electrical resistance (TEER) and paracellular permeability, and ELISA indicated that although IIL and AW ESP disrupted the cell monolayer integrity, autophagy induced by IIL and AW ESP also abolished and alleviated the ESP decreased-tight junctions expressions in Caco-2 monolayer, reduced the ESP-induced secretion of pro-inflammatory (TNF-α and IL-1β), and enhanced ESP-up-regulated production of anti-inflammatory cytokines (TGF-β).

**Conclusions:**

*T. spiralis* ESP-induced autophagy ultimately relieved and limited the damage of *T. spiralis* ESP to gut epithelial barrier, and ensured the *T. spiralis* survival and development in host gut mucosal epithelium.

## Author summary

*Trichinella spiralis* excretory-secretory proteins (ESP) disrupted host’s intestinal epithelial barrier and mediated worm invasion. Meanwhile, *T. spiralis* infection induced autophagy of murine intestinal epithelial cells. But, autophagy function in *T. spiralis* invasion and colonization in host remains unclear. The aim of this study was to investigate whether *T. spiralis* ESP induce enterocyte autophagy and whether the autophagy protects intestinal epithelial barrier from ESP-induced destruction. The results showed that IIL and AW ESP induced autophagy of Caco-2 and RAW264.7 cells via increasing RACK1 expression and intracellular Ca^2+^ concentration, and activating AMPK/mTOR pathway. Although IIL and AW ESP disrupted the cell monolayer integrity, the autophagy induced by IIL and AW ESP also abolished and alleviated the ESP decreased-tight junctions expressions in Caco-2 monolayer, reduced the ESP-induced secretion of pro-inflammatory (TNF-α and IL-1β), and enhanced ESP-up-regulated production of anti-inflammatory cytokines (TGF-β). Therefore, ESP-induced autophagy ultimately relieved and limited the *T. spiralis* ESP damage to gut epithelial barrier, and ensured the *T. spiralis* survival and development in host gut mucosal epithelium.

## Introduction

Trichinellosis is a zoonotic parasitic disease caused by infection with *Trichinella spiralis.* Mammals including humans, pigs and mice become infected with *T. spiralis* by ingesting animal meat containing the encapsulated muscle larvae (ML) in skeletal muscles. After the infected meat is ingested and digested, and the ML are isolated from their capsules in the stomach, they enter small intestine and develop into intestinal infectious larvae (IIL). The IIL invade intestinal epithelial cells (IECs) and develop into adult worms (AW) within intestinal epithelial niches. The adult worms mate and produce the newborn larvae (NBL). The NBL eventually reach skeletal muscles throughout the body via the bloodstream to form new encapsulated ML. Therefore, IIL and AW are two critical intestinal stages in *T. spiralis* infection [1,2]

Previous studies have showed that *T. spiralis* larval invasion of the host intestinal mucosa through disrupting the IEC tight junctions (TJs) and damaging integrity of intestinal epithelial barrier by its excretory secretory proteins (ESP) [3–6]. However, *T. spiralis* may also have mechanisms to limit the destruction of host intestine to ensure its survival itself in addition to damaging intestinal epithelium to facilitate larval invasion [7]. *T. spiralis* infection induced the autophagy of IECs [8]. Under the conditions of intestinal pathogen infection, autophagy typically plays a role in maintaining intestinal epithelial cell homeostasis and barrier integrity [9].

Autophagy is a complex self-degradation process, which delivers cytoplasmic components, organelles, and infectious agents to lysosomes for degradation. As a highly conserved mechanism, autophagy exists in all eukaryotic cells and participates in maintaining the normal physiological functions of the organism [10]. Autophagy plays a crucial role in coping with various stressors, including hypoxia, infection, endoplasmic reticulum stress, tissue remodeling, degradation of cellular debris, renewal of damaged organelles, tumor suppression, immune responses, and cell death. It is essential for maintaining cellular homeostasis. In small intestine, autophagy contributes significantly to regulation of inflammatory response, maintenance of intestinal barrier integrity, and homeostasis of gut mucosal environment [11].

Autophagy is regulated by multiple factors. The mechanistic target of rapamycin (mTOR) is a core regulatory factor [12]. mTOR inhibits autophagy initiation by directly phosphorylating the UNC-51-like autophagy activating kinase 1 (ULK1) complex. When mTOR is inhibited, the autophagy process is activated. Activation of AMP-activated protein kinase (AMPK) serves as an upstream regulator of the mTOR pathway. Increased AMPK activity leads to decreased mTOR activity, thereby initiating autophagy [13]. A 3-benzyl-5-((2-nitrophenoxy) methyl)-dihydrofuran- 2(3H)-one (3BDO) is a new mTOR activator which can also inhibit autophagy [14].

Previous studies showed that *T. spiralis* infection induced the IEC autophagy [8]. However, the role of autophagy in *T. spiralis* infection and the underlying mechanisms remain unclear. Autophagy enhances the tight junctions (TJs) barrier function of the IECs by promoting the expression of TJs proteins and their distribution to the cell surface [15,16]. Autophagy also prevents cytokine-mediated disruption of intestinal epithelial barrier by regulating cytokine expression. The regulatory role of autophagy on intestinal inflammation has been confirmed [17,18].

Caco-2 cell is a human colon carcinoma cell line, and is commonly used to simulate the characteristics and functions of human small intestinal epithelial cell layer [19]. However, Caco-2 cells alone cannot fully represent the cellular interactions occurring in the *in vivo* intestinal environment. By co-culturing the IECs with immune cells, a microenvironment resembling the *in vivo* intestinal mucosal barrier (composed of the epithelial layer and lamina propria immune cells) is constructed to recapitulate the physical contact and signaling communication between IECs and immune cells [20]. This approach is frequently used to investigate the association between epithelial barrier damage and immune responses in intestinal inflammation [21,22]. The macrophage RAW264.7 cells are widely used as an *in vitro* model for studying immunomodulatory substances [23,24]. The Caco-2/RAW264.7 co-culture model has been used to evaluate the regulatory effects of immune cell activities on intestinal epithelium barrier integrity and function [25,26].

The aim of this study was to investigate whether *T. spiralis* ESP induce enterocyte autophagy and whether autophagy protects intestinal epithelial barrier. The results will provide new perspectives to understand the mechanisms of *T. spiralis* colonization and development in intestine epithelium and to elucidate the interaction between *T. spiralis* and host intestine epithelium.

## Materials and methods

### Ethics statement

This study was performed in the light of National Guidelines for Experimental Animal Welfare (Minister of Science and Technology, People’s Republic of China, 2006). All animal experiments in this study were approved by the institutional Life Science Ethics Committee of Zhengzhou University (No. ZZUIRB GZR 2021–0044).

### *Trichinella* and experiment animals

The species of *Trichinella* spp. used in this experiment was *T. spiralis* isolate (ISS534) obtained from an infected domestic pig in Nanyang, Henan Province of China [27]. Female BALB/c mice aged 4–6 weeks old were purchased from Henan Experimental Animal Center.

### Chemicals and antibodies

Gut epithelial receptor RACK1 inhibitor Harringtonolide (HO) was obtained from MedChemExpress (New Jersey, USA). AMPK pathway inhibitor Dorsomorphin dihydrochloride (DD) and mTOR activator 3BDO were purchased from TargetMol (Massachusetts, USA) [28].

The primary antibodies used in this study include antibodies against ZO-1, RACK1, GAPDH and β-actin from Servicebio (Wuhan, China); Occludin and Claudin-1 from Thermo Fisher Scientific (Massachusetts, USA); p-AMPK and AMPK from HuaBio (Hangzhou, China), mTOR from Proteintech (Wuhan, China); p-mTOR from CST (Massachusetts, USA); Beclin-1 from Abcam (Cambridge, UK), p-62 from Sangon (Shanghai, China). The secondary antibodies used in this study include HRP-goat anti-mouse IgG and HRP-goat anti-rabbit IgG conjugate from Sangon (Shanghai, China), Cy3-goat anti-rabbit IgG from Servicebio (Wuhan, China).

### Worm collection and ES antigen preparation

The IIL and AW are two important intestinal stages during *T. spiralis* infection, and the protein components of IIL and AW ESP are significantly different [29], hence, both IIL and AW ESP are used in the present study.

Mice infected with *T. spiralis* were sacrificed at 6 hours post infection (hpi) and 3 days post infection (dpi) to collect the IIL and AW, respectively [5]. IIL and AW were cultivated in RPMI-1640 at 5000 worm/ml medium for 18 h at 37 °C and 5% CO_2_. The ESPs were prepared as reported before. The culture supernatant was concentrated by using Amicon Ultra-3 centrifugal filtration device (MW cut-off value: 3 kDa), and centrifuged at 4 °C, 5000 × *g*. The IIL and AW ESP were collected and stored at −80 °C till use [30–32].

### Cell culture and Caco-2/RAW264.7 co-culture

Caco-2 cells were purchased from the Cell Resource Center of Shanghai Institute of Biological Sciences, Chinese Academy of Sciences. Caco-2 cells were cultivated in Dulbecco’s Modified Eagle Medium (DMEM) (Servicebio, Wuhan, China), which was supplemented with 10% fetal bovine serum (FBS) (Biological Industries, Israel), 100 U/mL penicillin, 0.1 mg/mL streptomycin and 100 mM nonessential amino acids (Solarbio, Beijing, China). The cells were inoculated in T25 flasks (NEST, Wuxi, China) and cultured for 7 d. After being digested by trypsin (Solarbio, Beijing, China), the cells were again cultured at 37 °C and 5% CO_2_ for 7 d to converge on a cover slide in a 24-wells culture plate [5].

The RAW264.7 macrophages were expanded in DMEM (Servicebio, Wuhan, China) containing 5% FBS (Biological Industries, Israel), 100 U/mL penicillin, and 0.1 mg/mL streptomycin. They were then seeded into 24-well plates and cultured at 37 °C with 5% CO₂ for 3 d to confluence [23].

To investigate the role of *T. spiralis* ESP (TsESP) induced-autophagy in maintaining the integrity of Caco-2 cell monolayers during TsESP-triggered inflammatory responses, a Caco-2/RAW264.7 co-culture system was established. Briefly, Trans-well inserts with 0.4 μm pore size, which was permeable to the autophagy inhibitor 3BDO, were seeded with Caco-2 cells. The inserts were placed into 24-well plates pre-seeded with RAW264.7 cells with 80% confluence. The autophagy inhibitor 3BDO (60 μM) was added to the upper chamber [26]. After incubation for 24 h, IIL ESP (15 μg/ml) or AW ESP (15 μg/ml) was respectively added to the lower chamber and further incubated for 12 h [22].

### Western blot analysis

To assess the expression level of TJs and autophagy-related proteins of Caco-2 cells treated with IIL and AW ESP, Western blot analysis was performed as described before [7]. Briefly, protein samples were separated on SDS-PAGE with 6% separation gel, subsequently transferred onto the 0.22 μm pore size polyvinylidene fluoride (PVDF) membranes (Millipore, USA) at 0.25 A for 90 min in a wet transfer cell (Bio-Rad, USA) [33]. The membrane was blocked by 5% skim milk at room temperature for 2 h, cropped into strips, and the strips were incubated with primary antibodies against ZO-1 (1:1 000), Occludin (1:125), Claudin-1 (1:125), p-mTOR (1:1000), mTOR (1:800), p-AMPK (1:200), AMPK (1:200), p-62 (1:300), Beclin-1 (1:2 000), GAPDH (1:1 000) and β-Actin (1:1 000) at 4 °C overnight. After washes, the strips was incubated with HRP-goat anti-rabbit IgG (1:10 000) or HRP-goat anti-mouse IgG (1:10 000) at room temperature for 2 h. Color development of the strips was performed by the enhanced chemiluminescence (ECL) kit (Meilunbio, Shanghai, China) and the relative expression level was determined with Image J software [34].

### Intracellular Ca^2+^ concentration assay

Normal Caco-2 cells and the cells treated with the RACK1 inhibitor HO (40 μM, 24 h) were separately stimulated with IIL ESP (15 μg/ml, 12 h) or AW ESP (15 μg/ml, 12 h) [28]. The intracellular Ca^2+^ concentration was assayed using the Fluo-4AM Ca^2+^ fluorescent probe (MA0196, Dalian Meilun) [35,36].

### Real-time quantitative PCR (qPCR) assay

To investigate whether TsESP regulate the transcription levels of TJs and cytokines in Caco-2 cells or RAW264.7 cells, qPCR was conducted as previously reported [37,38]. Total RNAs were extracted from the cells treated with TRIzol (Sangon, Shanghai, China). The RNAs were reverse-transcribed into cDNA using Evo M-MLV RT Master Mix for qPCR (Accurate Biology, Hunan, China) as templates for qPCR. qPCR was performed by using Universal Blue Probe qPCR Master Mix (Servicebio, Wuhan, China) to amplify the cDNA. Transcription levels of the TJs (ZO-1, Occludin and Claudin-1), and cytokines (TNF-α, IL-1β, TGF-β, IL-10) were ascertained by qPCR with specific primers (Table 1) using Applied Biosystems 7500 Fast Real-Time PCR System. Relative transcription level of TJs and cytokines was normalized by subtracting the transcription of a housekeeping gene glyceraldehyde-3-phosphate dehydrogenase (GAPDH), and was calculated with the 2^−ΔΔCt^ method as previously reported [39–41].

**Table 1:** Primer sequences of TJs and cytokines in qPCR assays.

Gene names	Sequence (5′-3′)	GenBank no.
ZO-1 (Human)	F: CGGTCCTCTGAGCCTGTAAG	NM_001330239.4
	R: GGATCTACATGCGACGACAA	
Occludin (Human)	F: ATGGCAAAGTGAATGACAAGCGG	XM_026274194.1
	R: CTGTAACGAGGCTGCCTGAAGT	
Claudin-1 (Human)	F: GTCTTTGACTCCTTGCTGAATCTG	NM_021101.5
	R: CACCTCATCGTCTTCCAAGCAC	
TNF-α (Human)	F: CTCTTCTGCCTGCTGCACTTTG	NM_000594.4
	R: ATGGGCTACAGGCTTGTCACTC	
IL-1β (Human)	F: CCACAGACCTTCCAGGAGAATG	NM_000576.3
	R: GTGCAGTTCAGTGATCGTACAGG	
TGF-β (Human)	F: TACCTGAACCCGTGTTGCTCTC	XM_011527242.3
	R: GTTGCTGAGGTATCGCCAGGAA	
IL-10 (Human)	F: CCACAGACCTTCCAGGAGAATG	NM_000576
	R: GTGCAGTTCAGTGATCGTACAGG	
GAPDH (Human)	F: TGTGTCCGTCGTGGATCTGA	NM_002046.7
	R: TTGCTGTTGAAGTCGCAGGAG	
TNF-α (Mouse)	F: CCCTCACACTCAGATCATCTTCT	NM_013693.3
	R: GCTACGACGTGGGCTACAG	
IL-1β (Mouse)	F: AGCTCTCCACCTCAATGGAC	NM_008361.4
	R: ATCATTGCGTGGGATCTTGA	
TGF-β (Mouse)	F: AGCAACAATTCCTGGCGTTACCT	NM_011577.2
	R: CCTGTATTCCGTCTCCTTGGTTCA	
IL-10 (Mouse)	F: CCCTTTGCTATGGTGTCCTT	NM_010548.2
	R: TGGTTTCTCTTCCCAAGACC	
GAPDH (Mouse)	F: GGTTGTCTCCTGCGACTTCA	NM_001411840.1
	R: TGGTCCAGGGTTTCTTACTCC	

### Assay of trans-epithelial electrical resistance (TEER) and paracellular permeability

To evaluate the integrity of intestinal epithelial barrier, TEER was measured. Caco-2 cells were seeded into Trans-well inserts (6.5 mm in diameter, 0.4 µm pore size; BIOFIL, China) and cultured until confluence was achieved. The TEER was subsequently measured using a Millicell-ERS volt-ohmmeter (Millipore, USA), with the resistance value calculated as: (measured value - blank value) × membrane base area [4,42]. Once the TEER value was stabilized (above 400 Ω·cm^2^), 15 µg/ml of IIL or AW ESP was added to the apical side of the Caco-2 monolayer. The cells were then incubated at 37°C for 12 h, and then TEER was measured again. The TEER value was normalized to its initial value prior to treatment and expressed as a percentage of the initial TEER [7].

The paracellular permeability of Caco-2 monolayer was assessed using a 4 kDa FITC-dextran (FD-4, Sigma, USA) diffusion assay [43,44]. Caco-2 cells were seeded into a Trans-well insert and cultured to confluence. The cells were first incubated with 10 µM dorsomorphin dihydrochloride (DD, an AMPK inhibitor; TargetMol, USA) at 37 °C for 24 h, followed by incubation with IIL or AW ESP at 37 °C for an additional 24 h. Subsequently, 0.5 mg/ml FD-4 dissolved in the medium was added to the apical compartment of the monolayer. Concurrently, medium lacking FD-4 was added to the basal compartment and incubated at 37 °C for 2 h. The medium in basal compartment was then collected, and the fluorescence intensity was measured at an excitation wavelength of 485 nm and an emission wavelength of 520 nm using a microplate reader (Molecular Devices, USA) [28,45,46].

### Indirect immunofluorescence test (IIFT)

IIFT was used to assess the expression of TJs (ZO-1, Occludin and Claudin-1) in Caco-2 cells. Caco-2 monolayers were fixed with 4% paraformaldehyde and then incubated with the primary antibody (1:500 for anti-ZO-1; 1:160 for anti-Occludin; 1:50 for anti-Claudin-1) overnight at 4 °C. Subsequently, the monolayers were incubated with Cy3-goat anti-rabbit IgG for 2 h at room temperature. The cell nuclei were stained blue with 4’,6-Diamidino-2-phenylindole (DAPI). The slides were observed and photographed under the fluorescence microscope (Olympus, Japan) [7,28].

### ELISA determination of cytokines

To ascertain the levels of cytokines produced by Caco-2 cells and RAW264.7 cells, an ELISA assay was performed [47]. After the cells were treated, the cell culture supernatants were collected, centrifuged at 500 × *g* for 5 min, and they were used as test samples. For detecting murine TNF-α and IL-10 in RAW264.7 cells, antibodies against TNF-α and IL-10 (Biolegend, San Diego, USA) were used. For detecting human TNF-α, IL-1β, TGF-β and IL-10, and mouse IL-1β and TGF-β, the ELISA kits (DaYou, Beijing, China) were used respectively. The ELISA determination of cytokines was carried out according to the instructions and operation guides of these kits.

### Statistical analysis

All data in this research are presented as means ± standard deviation (SD) and analyzed with SPSS 27.0 software. Data were analyzed by two tailed Student t-test or Mann-Whitney U test. Statistical significance was considered when *P* < 0.05.

## Results

### TsESP induces autophagy through AMPK/mTOR pathway in Caco-2 cells

**Fig 1:**
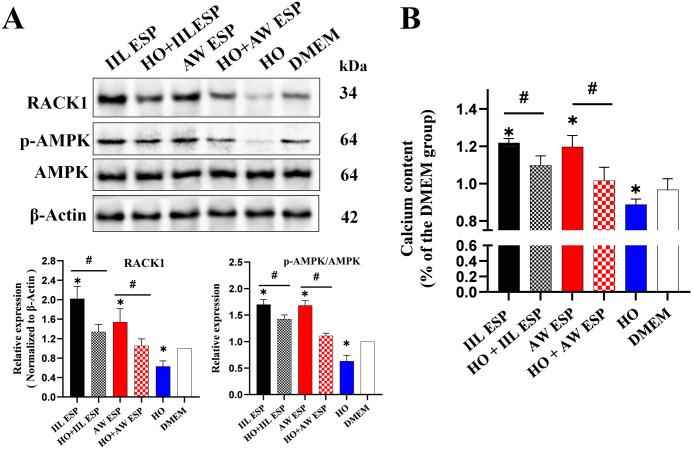
TsESP activated AMPK pathway via increasing RACK1 expression in Caco-2 cells, and increased intracellular Ca² ^+^ concentration. **A**: Both IIL and AW ESP increased the RACK1 expression in Caco-2 cells and activated the AMPK pathway. When RACK1 was inhibited, the IIL ESP activated-AMPK was also inhibited. **B**: IIL and AW ESP increased the intracellular Ca^2+^ concentration in Caco-2 cells by increasing RACK1 expression. When RACK1 was inhibited, intracellular Ca^2+^ concentration was decreased. Data are presented as mean ± SD of three independent assays. *Compared with the DMEM group, *P* < 0.05; # compared with the IIL or AW ESP group, *P* < 0.05.

Stimulation of Caco-2 cells with IIL or AW ESP increased the expression levels of RACK1 (*t*_IIL ESP_ = 7.035, *P* = 0.02; *U*_AW ESP_ = 6, *P* = 0.014), and p-AMPK/AMPK (*t*_IIL ESP_ = 13.434, *P* < 0.0001; *t*_AW ESP_ = 13.381, *P* < 0.0001), activated the AMPK signaling pathway. Treatment of Caco-2 cells with the RACK1 receptor inhibitor HO decreased the expression level of both RACK1 (*t* = -5.754 *P* = 0.005) and p-AMPK/AMPK (*U* = 6, *P* = 0.014), inhibiting *t*he AMPK signaling pathway. Pretreatment of Caco-2 cells with HO and then incubation with IIL or AW ESP significantly reduced the expression levels of RACK1 (*t*_IIL ESP_ = 4.016, *P* = 0.016; *U*_AW ESP_ = 6, *P* = 0.014) and p-AMPK/AMPK (*t*_IIL ESP_ = 4.03, *P* = 0.016; *t*_AW ESP_ = 9.906, *P* = 0.002), compared *t*o *t*he only ESP treatment group (Fig 1A). These results showed that ESP activates the AMPK pathway in Caco-2 cells via RACK1 up-regulation.

Similarly, IIL and AW ESP increased intracellular Ca^2+^ concentration (*t*_IIL ESP_ = 8.020, *P* = 0.001; *t*_AW ESP_ = 4.864, *P* = 0.003); while the HO decreased intracellular Ca^2+^ concentration (*t* = 3.594, *P* = 0.018), and inhibited the AMPK pathway. When the cells pretreated with HO were stimulated with IIL or AW ESP, Ca^2+^ levels were significantly lower than the only TsESP group (*t*_IIL ESP_ = 4.386, *P* = 0.011; *t*_AW ESP_ = 5.832, *P* = 0.009) (Fig 1B). Therefore, the findings indicated that ESP activated the AMPK pathway in Caco-2 cells by upregulating RACK1 expression, which increased intracellular Ca^2+^ concentration.

**Fig 2:**
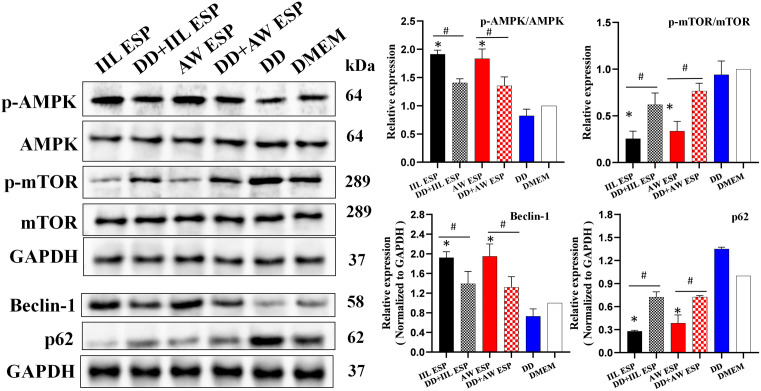
TsESP induces Caco-2 cell autophagy through activating AMPK/mTOR signaling pathway. IIL and AW ESP increased the p-AMPK/AMPK expression while decreased p-mTOR/mTOR expression, activating the AMPK/mTOR signaling pathway. They also increased the expression Beclin-1 level and decreased the p62 amount, inducing Caco-2 cell autophagy. When AMPK pathway was inhibited, the mTOR pathway activation induced by IIL and AW ESP was also reduced, the Beclin-1 expression was decreased, the p62 level was increased and Caco-2 cell autophagy caused by IIL ESP and AW ESP was inhibited. Data are presented as mean ± SD of three independent assays. *Compared with the DMEM group, *P* < 0.05; ^#^compared with the IIL ESP or AW ESP group, *P* < 0.05.

Western blot results showed that when Caco-2 cells were stimulated by IIL or AW ESP, relative expression levels of p-AMPK/AMPK were significantly increased (*t*_IIL ESP_ = 22.586, *P* = 0.002; *t*_AW ESP_ = 8.437, *P* = 0.014). Concurrently, a notable decrease of p-mTOR/mTOR expression levels was also detected (*t*_IIL ESP_ = -15.967, *P* < 0.0001; *t*_AW ESP_ = -11.122, *P* = 0.008), suggesting the activation of the AMPK/mTOR signaling pathway. Simultaneously, an upregulation of expression level of autophagy-related protein Beclin-1 (*t*_IIL ESP_ = 13.441, *P* = 0.005; *t*_AW ESP_ = 6.852, *P* = 0.003) was accompanied by a decrease in the amount of the autophagic substrate p62 (*t*_IIL ESP_ = -113.362, *P* < 0.0001; *t*_AW ESP_ = -10.125, *P* = 0.001). Collectively, these results implied that both IIL ESP and AW ESP were capable of inducing autophagy of Caco-2 cells. To further investigate the underlying mechanism, the inhibitor Dorsomorphin (DD) of AMPK pathway was used in Caco-2 cells. After treatment with DD, the IIL and AW ESP-induced decrease of p-mTOR/mTOR expression level was significantly reversed (*t*_IIL ESP_ = -4.259, *P* = 0.013; *t*_AW ESP_ = -5.661, *P* = 0.005). Similarly, the upregula*t*ion of autophagy-related protein Beclin-1 expression level was also diminished (*t*_IIL ESP_ = 3.329, *P* = 0.029; *t*_AW ESP_ = 3.325, *P* = 0.029), and the consumption of autophagic substrate p62 was reduced (*t*_IIL ESP_ = -11.092, *P* < 0.0001; *t*_AW ESP_ = -5.527, *P* = 0.028). These results clearly indicated that the inhibition of AMPK pathway effectively mitigated the Caco-2 cell autophagy triggered by IIL and AW ESP. Therefore, it was concluded that IIL and AW ESP induced Caco-2 cells autophagy through the activation of AMPK/mTOR signaling pathway (Fig 2).

Moreover, after Caco-2 cells were incubated with 15 μg/ml IIL ESP, the content of an autophagy marker Beclin-1 and an autophagy substrate p62 was ascertained by Western blot. The results showed that the Beclin-1 expression level was significantly increased at 12 h post incubation, but obviously decreased at 24 h post incubation (*t*_12 h =_ -6.046. *P* *<* 0.05; *t*_24 h =_ -4.717. *P* < 0.01), and had no significant difference from pre-incubation level at 36 h post incubation (*P* > 0.05). The abundance of the autophagy substrate p62 was distinctly decreased at 12 h post incubation and continued to decline at 24–36 h after incubation (*t*_12 h =_ 8.784. *P* < 0.01; *t*_24 h =_ 36.976. *P* < 0.0001; *t*_36 h =_18.689, *P* < 0.01) (S1 Fig). These findings indicated tha*t* IIL ESP induced Caco-2 cell autophagy, the autophagy markers exhibited a time-dependent kinetic change: Beclin-1 level was first elevated and then declined, and the autophagy substrate p62 content was gradually reduced along with the extension of incubation.

### TsESP induced RAW264.7 cell autophagy through AMPK/mTOR pathway

**Fig 3:**
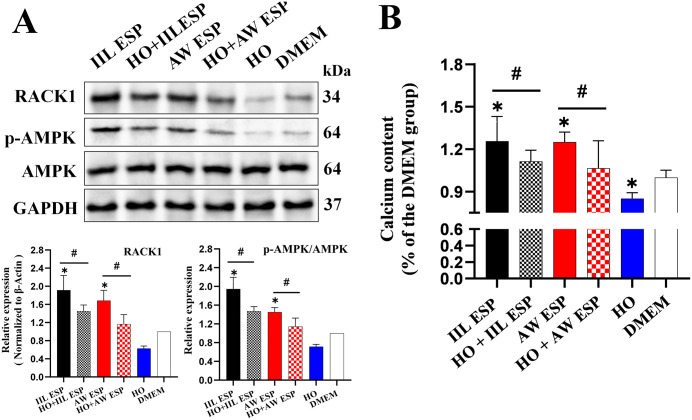
TsESP activated AMPK via increasing RACK1 expression in RAW 264.7 cells, and elevated the intracellular Ca² ^+^ concentration. **A**: Both IIL and AW ESP increased the RACK1 expression in RAW264.7 cells and activated the AMPK pathway. When RACK1 was inhibited, the IIL and AW ESP-induced AMPK activation was also inhibited. **B**: IIL and AW ESP increased the intracellular Ca^2+^ concentration in RAW264.7 cells by increasing RACK1 expression. When RACK1 was inhibited, intracellular Ca^2+^ concentration was also decreased. Data are presented as mean ± SD of three independent assays. *Compared with the DMEM group, *P* < 0.05; # compared with the IIL ESP or AW ESP group, *P* < 0.05.

When RAW264.7 cells were incubated with IIL ESP (10 μg/ml, 12 h) or AW ESP (10 μg/ml, 12 h), the expression of RACK1 and p-AMPK/AMPK was significantly increased (RACK1: *t*_IIL ESP_ = 4.888, *P* = 0.008; *t*_AW ESP_ = 5.237, *P* = 0.035; p-AMPK/AMPK: *t*_IIL ESP_ = 6.547, *P* = 0.023; *t*_AW ESP_ = 8.253, *P =* 0.014), thereby activating the AMPK signaling pathway. Treatment with RACK1 inhibitor HO obviously decreased expression levels of RACK1 (*t* = -11.87, *P* = 0.007) and p-AMPK/AMPK (*t* = -10.147, *P* = 0.001), inhibiting the AMPK pathway. Pretreatment with HO and then incubation with IIL or AW ESP reduced the expression of RACK1 (*U*_IIL ESP_ = 6, *P* = 0.014; *U*_AW ESP_ = 2.915, *P* = 0.043) and p-AMPK/AMPK (*t*_IIL ESP_ = 3.063, *P* = 0.038; *U*_AW ESP_ = 6, *P* = 0.014), compared to only TsESP treatment (Fig 3A). These results indicated that TsESP activates the AMPK pathway in RAW264.7 cells via up-regulating RACK1 expression.

Similarly, IIL and AW ESP treatment significantly increased intracellular Ca^2+^ concentration (*t*_IIL ESP_ = 8.020, *P* = 0.001; *t*_AW ESP_ = 4.864, *P* = 0.003), while HO treatment decreased Ca^2+^ levels (*t* = -3.594, *P* = 0.018), thereby inhibiting the AMPK pathway. When HO-pretreated cells were stimulated with IIL or AW ESP, Ca^2+^ levels were significantly lower than in the TsESP alone group (*t*_IIL ESP_ = 4.386, *P* = 0.011; *t*_AW ESP_ = 5.832, *P* = 0.009) (Fig 3B). These findings suggest that TsESP activates the AMPK pathway in RAW264.7 cells by up-regulating RACK1, which in turn increased intracellular Ca^2+^ concentration.

**Fig 4:**
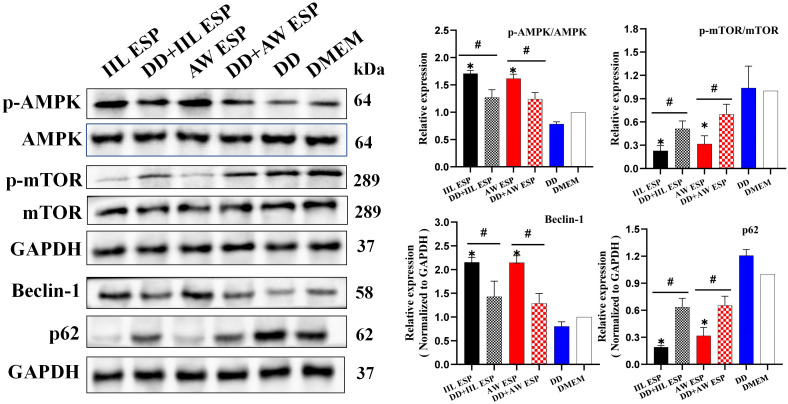
TsESP induced autophagy through the AMPK/mTOR pathway in RAW264.7 cells. IIL and AW ESP increased p-AMPK/AMPK expression and decreased p-mTOR/mTOR expression, activating the AMPK/mTOR signaling pathway. They also increased Beclin-1 expression and decreased the amount of p62, inducing autophagy in RAW264.7 cells. When AMPK was inhibited, the mTOR pathway activation induced by IIL and AW ESP was also attenuated, the Beclin-1 expression was decreased, the p62 level was increased, IIL and AW ESP-induced RAW264.7 cells autophagy was inhibited. Data are presented as mean ± SD of three independent assays. *Compared with the DMEM group, *P* < 0.05; # compared with the IIL ESP or AW ESP group, *P* < 0.05.

When RAW264.7 cells were treated with IIL or AW ESP, the p-AMPK/AMPK expression was significant increased (*t*_IIL ESP_ = 20.856, *P* < 0.001; *t*_AW ESP_ = 13.58, *P* < 0.001), and p-mTOR/mTOR expression was no*t*ably decreased (*t*_IIL ESP_ = -20.359, *P* < 0.001; *t*_AW ESP_ = -11.384, *P* < 0.001), suggesting the activation of AMPK/mTOR signaling pathway. Simultaneously, expression level up-regulation of autophagy-related protein Beclin-1 (*t*_IIL ESP_ = 19.689, *P* < 0.001; *t*_AW ESP_ = 4.193, *P* = 0.014) was accompanied by a decrease of the amount of autophagy substrate p-62 (*t*_IIL ESP_ = -84.187, *P* < 0.001; *t*_AW ESP_ = -13.023, *P* = 0.006) (Fig 4). Collectively, these results implied that both IIL and AW ESP were capable of inducing RAW264.7 cell autophagy. To further clarify the possible mechanism, the AMPK pathway in RAW264.7 cells was suppressed by the AMPK inhibitor DD. After treatment with DD, the IIL and AW ESP-decreased p-mTOR/mTOR expression level was significantly restored and increased. Similarly, when RAW264.7 cells were pretreated by DD, the up-regulation of autophagy-related protein Beclin-1 expression level was also reduced (*t*_IIL ESP_ = 1.371, *P* = 0.024; *t*_AW ESP_ = 5.855, *P* = 0.004), and the autophagy substrate p-62 was increased (*t*_IIL ESP_ = -7.849, *P* = 0.001; *t*_AW ESP_ = -4.243, *P* = 0.014). These findings clearly indicated that the AMPK inhibition effectively alleviated the RAW264.7 cell autophagy induced by IIL and AW ESP. Therefore, it was concluded that IIL and AW ESP induced RAW264.7 cell autophagy via activating the AMPK/mTOR signaling pathway.

### Autophagy inhibitor 3BDO aggravated TsESP-disrupted Caco-2 monolayer integrity

**Fig 5:**
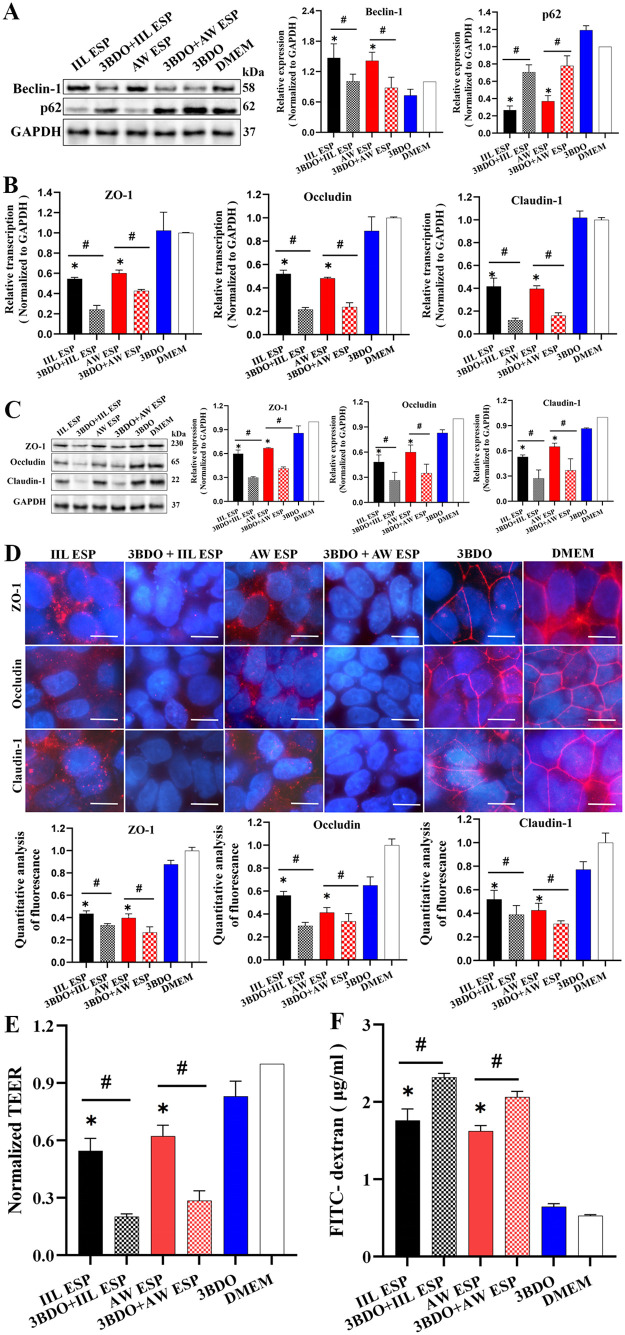
Autophagy inhibitor 3BDO exacerbated TsESP-disrupted Caco-2 monolayer barrier. **A:** Treatment of Caco-2 cells with autophagy inhibitor 3BDO reduced TsESP increased-Beclin-1expression and elevated TsESP-reduced p62. 3BDO inhibited Caco-2 cell autophagy caused by IIL and AW ESP. **B:** qPCR analysis of TJs mRNA levels (ZO-1, Occludin and Claudin-1) in Caco-2 cells treated with 3BDO and IIL or AW ESP **C:** Western blotting of TJs protein levels (ZO-1, Occludin and Claudin-1) in Caco-2 cells treated with 3BDO and IIL or AW ESP. **D:** IIFT analysis of TJs protein levels (ZO-1, Occludin and Claudin-1) in Caco-2 cells treated with 3BDO and IIL or AW ESP. Scale bars: 10 μm. **E:** trans-epithelial electrical resistance (TEER) of Caco-2 monolayers treated with 3BDO and IIL or AW ESP. **F:** paracellular permeability of Caco-2 monolayers treated with 3BDO and IIL or AW ESP. Data are presented as mean ± SD of three independent assays. *Compared with the DMEM group, *P* < 0.05; ^#^ compared with the only IIL ESP or AW ESP alone group, *P* < 0.05.

Normal Caco-2 cells and Caco-2 cells pretreated with autophagy inhibitor 3BDO at 60 μM for 24 h were incubated with 15 μg/ml IIL or AW ESP at 37 °C for 12 h. Western blots showed that IIL and AW ESP obviously up-regulated Beclin-1 expression (*U*_IIL ESP_ = 0, *P* = 0.037; *t*_AW ESP_ = 4.327, *P* = 0.049), and decreased the p-62 contents (*t*_IIL ESP_ = -27.176, *P* = 0.001; *t*_AW ESP_ = -17.043, *P* = 0.003), but autophagy inhibitor 3BDO abolished and reversed the effect of IIL and AW ESP, as manifested by the Beclin-1 expression was decreased (*t*_IIL ESP_ = 2.868, *P* = 0.046; *t*_AW ESP_ = -3.463, *P* = 0.026) and the p-62 content was increased (*t*_IIL ESP_ = -7.897, *P* = 0.001; *t*_AW ESP_ = -17.043, *P* = 0.003). The above results indicated that Caco-2 cell autophagy triggered by IIL and AW ESP could be inhibited by autophagy inhibitor 3BDO (Fig 5A).

The qPCR results showed that compared with normal Caco-2 cells, the IIL and AW ESP significantly reduced the mRNA levels of ZO-1 (*t*_IIL ESP_ = -72.551, *P* < 0.0001; *t*_AW ESP_ = -31.75, *P* < 0.0001), Occludin (*t*_IIL ESP_ = -36.99, *P* < 0.0001; *t*_AW ESP_ = 125.481, *P* < 0.0001), and Claudin-1 (*t*_IIL ESP_ = -19.234, *P* < 0.0001; *t*_AW ESP_ = -44.678, *P* < 0.0001) in Caco-2 cells. Inhibition of Caco-2 cell autophagy with inhibitor 3BDO further declined the IIL ESP-reduced mRNA levels of ZO-1, Occludin and Claudin-1 (*t*_ZO-1_ *=* 17.259*, P <* 0.0001; *t*_Occludin_ *=* 20.972*, P <* 0.0001; *t*_Claudin-1_ = 9.851, *P <* 0.0001), and 3BDO also further declined AW ESP-reduced mRNA levels of ZO-1, Occludin and Claudin-1 (*t*_ZO-1_ = 12.75, *P* < 0.0001; *t*_Occludin_ = 16.43, *P* < 0.0001; *t*_Claudin-1_ = 16.029, *P* < 0.0001) (Fig 5B).

Western blotting results revealed that compared with normal Caco-2 cells, the IIL and AW ESP obviously decreased the protein expressions of ZO-1 (*t*_IIL ESP_ = -15.393, *P* = 0.004; *t*_AW ESP_ = -76.692, *P* < 0.0001), Occludin (*t*_IIL ESP_ = -7.927, *P* = 0.016; *t*_AW ESP_ = -5.297, *P* = 0.034), and Claudin-1 (*t*_IIL ESP_ = -35.615, *P* = 0.001; *t*_AW ESP_ = -15.008, *P* = 0.004) in Caco-2 cells. Suppression of Caco-2 cell autophagy with inhibitor 3BDO further exacerbated and declined the IIL ESP-reduced TJs protein expression levels (*t*_ZO-1_ = 11.1116*, P =* 0.005*; t*_Occludin_ *=* 2.979*, P* = 0.041*; t*_Claudin-1_ = 4.343*, P =* 0.041); 3BDO also further aggravated and declined AW ESP-reduced TJs protein expression levels (*t*_ZO-1_ = 22.135, *P* < 0.0001; *t*_Occludin_ *=* 3.204, *P* = 0.033*; t*_Claudin-1_ = 3.414 *P =* 0.027) (Fig 5C).

The IIFT results showed that in normal Caco-2 cells (e.g., the DMEM group), the TJs (ZO-1, Occludin and Claudin-1) were distributed around the Caco-2 cell border; but after the cells were treated with IIL and AW ESP, the quantities of ZO-1 (*t*_IIL ESP_ = -25.354, *P* < 0.0001; *t*_AW ESP_ = -9.175, *P* = 0.007), Occludin (*t*_IIL ESP_ = -11.686, *P* < 0.0001; *t*_AW ESP_ = -7.405, *P* = 0.002) and Claudin-1 (*t*_IIL ESP_ = -7.5, *P* = 0.002; *t*_AW ESP_ = -12.477, *P* < 0.0001) around *t*he cells were obviously decreased, and the immunostaining of continuous cellular border was lost or interrupted, suggesting that ESP treatment led to the degradation of TJs in Caco-2 monolayer. Moreover, suppression of Caco-2 cell autophagy with inhibitor 3BDO further exacerbated and declined the IIL ESP-reduced TJs expression levels (*t*_ZO-1_ = 2.985, *P* = 0.041; *t*_Occludin_ = 6.155, *P* = 0.004; *t*_Claudin-1_ = 9.861, *P* = 0.001); 3BDO also fur*t*her aggravated and declined AW ESP-reduced TJs protein expression levels (*t*_ZO-1_ = 3.693, *P* = 0.021; *t*_Occludin_ = 3.045, *P* = 0.038; *t*_Claudin-1_ = 3.108, *P* = 0.036) (Fig 5D). The results indicated that Caco-2 cell autophagy induced by IIL and AW ESP relieved the TJs protein reduction caused by IIL and AW ESP themselves.

The results of TEER and paracellular permeability assay showed that after Caco-2 monolayers were treated by IIL and AW ESP, the TEER are obviously decreased (*t*_IIL ESP_ = -12.256, *P* = 0.007; *t*_AWESP_ = -11.438, *P* < 0.0001), and paracellular permeability (*t*_IIL ESP_ = 5.134, *P* = 0.002; *t*_AW ESP_ = 4.693, *P* = 0.004) were increased evidently. Moreover, inhibition of Caco-2 autophagy with 3BDO further exacerbated the TEER decrease (*t*_IIL ESP_ = 9.092, *P* = 0.001; *t*_AW ESP_ = 7.62, *P* = 0.002), and the increased paracellular permeability was further elevated (*t*_IIL ESP_ = -27.256, *P* < 0.0001; *t*_AW ESP_ = -10.862, *P* < 0.0001) (Fig 5E and 5F).

The findings indicated that IIL and AW ESP disrupted Caco-2 monolayer integrity, the autophagy inhibitor 3BDO further exacerbated the integrity damage to increase its permeability, suggesting that ESP-induced Caco-2 cell autophagy might alleviated the destruction of Caco-2 monolayer integrity caused by IIL and AW ESP, thereby partially protecting the monolayer barrier function.

### Autophagy alleviated the TsESP-induced inflammatory response in Caco-2 cells

To investigate the effects of IIL ESP and AW ESP on inflammatory response in Caco-2 cells, normal Caco-2 cells and Caco-2 cells pretreated with autophagy inhibitor 3BDO were respectively stimulated with IIL ESP or AW ESP for 12 h. Transcription levels of pro-inflammatory cytokines (TNF-α and IL-1β) and anti-inflammatory cytokines (TGF-β and IL-10) in Caco-2 cells were assessed by qPCR. The concentrations of pro-inflammatory cytokines (TNF-α and IL-1β) and anti-inflammatory cytokines (TGF-β and IL-10) in Caco-2 culture supernatants were measured by ELISA to assess the levels of these cytokines secreted by Caco-2 cells.

**Fig 6:**
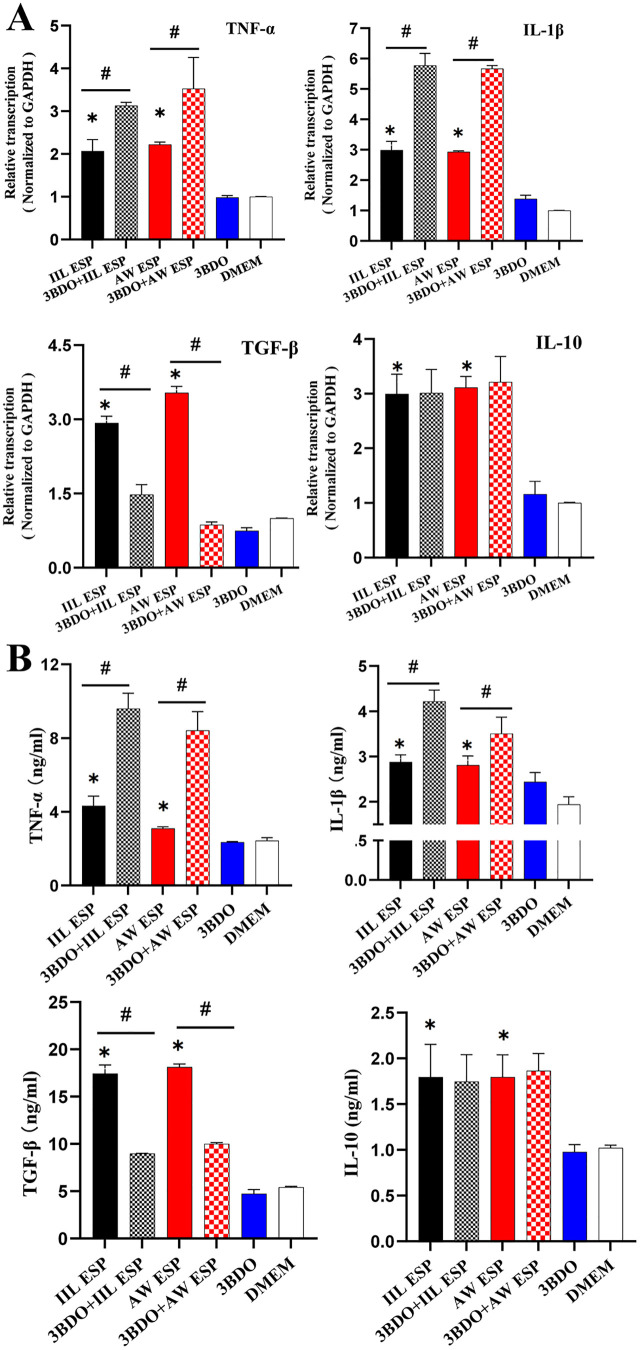
Expression levels of pro-inflammatory and anti-inflammatory cytokines in Caco-2 cells treated with IIL and AW ESP, and 3BDO. **A:** qPCR analysis of transcription level of pro-inflammatory and anti-inflammatory cytokines. IIL and AW ESP significantly increased transcription levels of pro-inflammatory (TNF-α and IL-1β) and anti-inflammatory cytokines (TGF-β and IL-10) in Caco-2 cells. Autophagy inhibitor 3BDO further elevated levels of TNF-α and IL-1β, while reducing TGF-β levels, but did not significantly affect the level of IL-10. **B:** ELISA determination of pro-inflammatory and anti-inflammatory cytokines. IIL and AW ESP significantly increased the secretion levels of pro-inflammatory (TNF-α, IL-1β) and anti-inflammatory cytokines (TGF-β, IL-10) in Caco-2 cells. 3BDO further elevated the levels of TNF-α and IL-1β, while reducing TGF-β levels, but did not significantly affect the IL-10 level. Data are presented as mean ± SD of three independent assays. ^*^Compared with the DMEM group, *P* < 0.05; ^#^ compared with the only IL ESP or AW ESP group, *P* < 0.05.

Results showed that IIL and AW ESP significantly up-regulated mRNA levels of both pro-inflammatory cytokines TNF-α and IL-1β (TNF-α: *t*_IIL ESP_ = -9.396, *P* < 0.001; *t*_AW ESP_ = 57.266, *P* < 0.001. IL-1β: *t*_IIL ESP_ = 17.092, *P* < 0.001; *t*_AW ESP_ = 160.619, *P* < 0.001). The mRNA levels of anti-inflammatory cytokines TGF-β and IL-10 in Caco-2 cells were obviously increased (TGF-β: *t*_IIL ESP_ = 34.4, *P* = 0.001; *t*_AW ESP_ = 47.973 *P* < 0.001; IL-10: *t*_IIL ESP_ = 13.746, *P* < 0.001; *t*_AW ESP_ = 25.886 *P* < 0.001). Furthermore, compared with the single ESP group, inhibition of Caco-2 cell autophagy with 3BDO further accentuated the increased mRNA levels of pro-inflammatory cytokines TNF-α and IL-1β(TNF-α: *t*_IIL ESP_ = -9.396, *P* = 0.007; *t*_AW ESP_ = -4.355 *P* = 0.007. IL-1β: *t*_IIL ESP_ = -14.025, *P* < 0.001; *t*_AW ESP_ = -65.04, *P* < 0.001), and alleviated the up-regulation of anti-inflammatory cytokines TGF-β (*t*_IIL ESP_ = 14.609, *P* < 0.001; *t*_AW ESP_ = 46.168, *P* < 0.001), while 3BDO had no significant effect on mRNA levels of IL-10 (Fig 6A). These findings indicated that IIL and AW ESP increased transcription levels of pro-inflamma*t*ory and anti-inflamma*t*ory cytokines of Caco-2 cells, while autophagy inhibitor 3BDO further elevated pro-inflammatory cytokines, but declined anti-inflammatory cytokine, suggesting that autophagy alleviated TsESP-induced inflammatory response in Caco-2 cells.

ELISA results revealed that IIL and AW ESP significantly increased the secretion of pro-inflammatory cytokines TNF-α and IL-1β in Caco-2 cells (TNF-α: *t*_IIL ESP_ = 7.021, *P* = 0.002; *t*_AW ESP_ = 6.365, *P* = 0.003; IL-1β: *t*_IIL ESP_ = 7.222, *P* = 0.002; *t*_AW ESP_ = 5.795, *P* = 0.004); and production of anti-inflammatory cytokines TGF-β and IL-10 was obviously increased (TGF-β: *t*_IIL ESP_ = 16.526, *P* = 0.004; *t*_AW ESP_ = 40.247, *P* < 0.001; IL-10: *t*_IIL ESP_ = 3.739, *P* = 0.02; *t*_AW ESP_ = 5.51, *P* = 0.029). Moreover, compared wi*t*h the ESP alone, inhibition of Caco-2 cell autophagy with 3BDO further elevated the secretion of TNF-α and IL-1β (TNF-α: *t*_IIL ESP_ = -13.434, *P* = 0.005; *t*_AW ESP_ = -8.955, *P* = 0.012; IL-1β: *t*_IIL ESP_ = -7.9, *P* = 0.001; *t*_AW ESP_ = -2.924, *P* = 0.043), while reduced the secretion of TGF-β (*t*_IIL ESP_ = 23.374, *P* = 0.002; *t*_AW ESP_ = 64.023, *P* < 0.001), and had no significant effect on the secretion of IL-10 (Fig 6B).

These findings demonstrated that IIL and AW ESP triggered an evident inflammatory response in Caco-2 cells, as demonstrated by obvious increased expression levels of both pro-inflammatory and anti-inflammatory cytokines; while autophagy inhibitor 3BDO further increased pro-inflammatory cytokine levels (TNF-α and IL-1β), but reduced anti-inflammatory cytokine TGF-β level, suggesting that IIL and AW ESP-induced autophagy alleviated Caco-2 cell inflammation reaction induced by themselves.

### Autophagy alleviated the TsESP-induced inflammatory response in RAW264.7 cells

**Fig 7:**
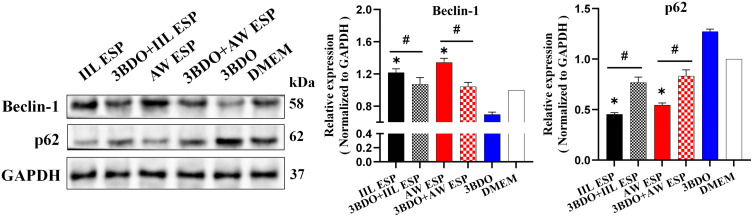
Western blotting of Beclin-1 and p62 protein expression in RAW264.7 cells treated with IIL and AW ESP and/or autophagy inhibitor 3BDO. IIL and AW ESP significantly up-regulated Beclin-1 expression and reduced p62 content; 3BDO declined Beclin-1 expression and increased p62 content, suggesting that 3BDO inhibited RAW264.7 cells autophagy caused by IIL and AW ESP. Data are presented as mean ± SD of three independent assays. *Compared with the DMEM group, *P* < 0.05; # compared with the IIL ESP or AW ESP group, *P* < 0.05.

Normal RAW264.7 cells and RAW264.7 cells pretreated with autophagy inhibitor 3BDO at 60 μM for 12 h were respectively stimulated with IIL or AW ESP at 10 μg/ml for 12 h. Western blot showed that IIL and AW ESP significantly up-regulated Beclin-1 expression (*t*_IIL ESP_ = 8.287, *P* = 0.014; *t*_AW ESP_ = 11.631, *P* = 0.007) and reduced p62 content (*t*_IIL ESP_ = -58.534, *P* < 0.0001; *t*_AW ESP_ = -40.978, *P* = 0.001); whereas autophagy inhibitor 3BDO declined Beclin-1 expression (*t*_IIL ESP_ = 3.935, *P* = 0.017; *t*_AW ESP_ = 7.098, *P* = 0.002) and increased p62 content (*t*_IIL ESP_ = -9.978, *P* = 0.001; *t*_AW ESP_ = -7.907, *P* = 0.001), in comparison with only IIL or AW ESP group (Fig 7). The results suggested that IIL or AW ESP induced RAW264.7 cell autophagy and the autophagy could be inhibited by 3BDO.

**Fig 8:**
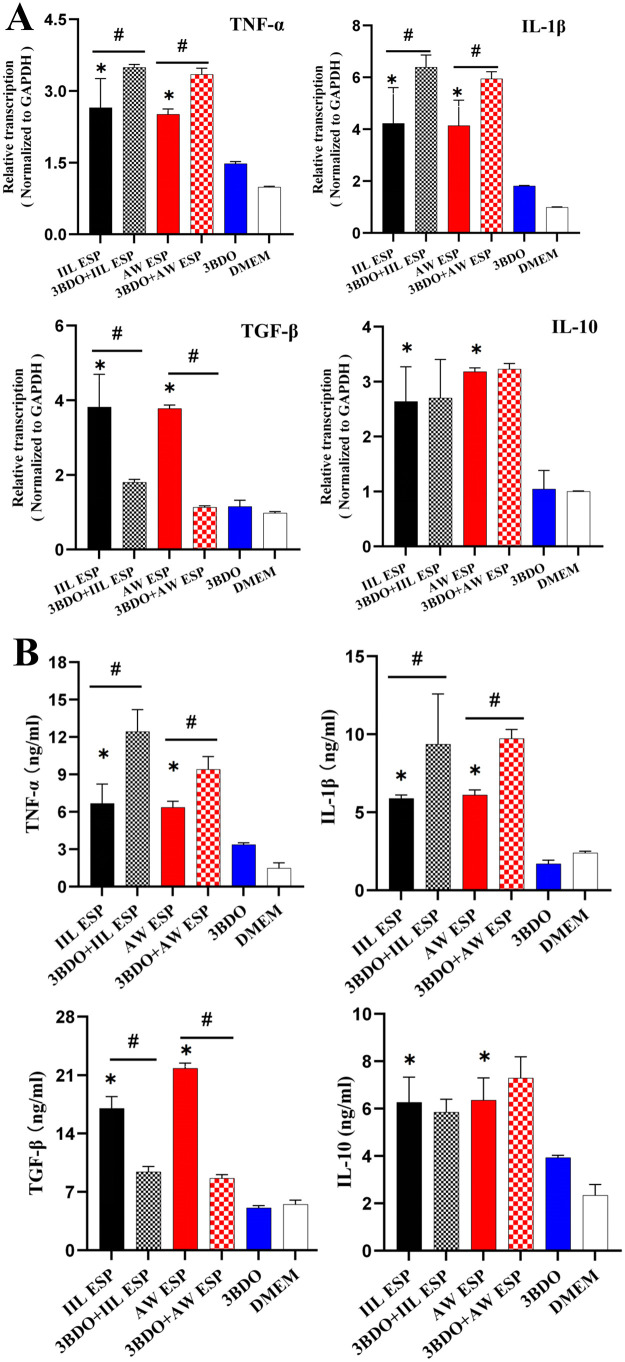
Transcription and expression levels of pro-inflammatory and anti-inflammatory cytokines in RAW264.7 cells treated with IIL and AW ESP, and 3BDO. **A:** qPCR analysis of transcription levels of pro-inflammatory and anti-inflammatory cytokines. IIL and AW ESP significantly increased the transcription levels of pro-inflammatory (TNF-α and IL-1β) and anti-inflammatory cytokines (TGF-β and IL-10) in RAW264.7 cells. Autophagy inhibitor 3BDO further elevated TNF-α and IL-1β mRNA levels, reduced TGF-β mRNA levels, but did not significantly affect the IL-10 mRNA level. **B:** ELISA determination of pro-inflammatory and anti-inflammatory cytokines. IIL and AW ESP significantly increased the secretion of pro-inflammatory (TNF-α, IL-1β) and anti-inflammatory cytokines (TGF-β and IL-10) in RAW264.7 cells. Autophagy inhibitor 3BDO further increased TNF-α and IL-1β secretion, reduce TGF-β secretion, but did not significantly affect the IL-10 level. Data are presented as mean ± SD of three independent assays. *Compared with the DMEM group, *P* < 0.05; ^#^ compared with the only IIL ESP or AW ESP group, *P* < 0.05.

To investigate the effects of IIL and AW ESP on inflammatory response in RAW264.7 cells, normal RAW264.7 and RAW264.7 cells pretreated with autophagy inhibitor 3BDO (60 μM for 24 h) were separately treated with IIL or AW ESP (10 μg/ml for 12 h). The transcription levels of pro-inflammatory (TNF-α and IL-1β) and anti-inflammatory cytokines (TGF-β and IL-10) in RAW264.7 cells were measured by qPCR. The concentrations of these inflammatory cytokines in RAW264.7 cell culture supernatants were measured by ELISA. The qPCR results showed that IIL and AW ESP significantly up-regulated transcriptional levels of pro-inflammatory cytokines TNF-α and IL-1β in RAW264.7 cells (TNF-α: *t*_IIL ESP_ = 5.776, *P* = 0.002; *t*_AW ESP_ = 34.062, *P* < 0.001; IL-1β: *t*_IIL ESP_ = 5.776, *P* = 0.002; *t*_AW ESP_ = 7.856, *P* = 0.001). IIL and AW ESP also obviously increased *t*ranscription levels of anti-inflammatory cytokines TGF-β and IL-10 (TGF-β: *t*_IIL ESP_ = 7.947, *P* = 0.001; *t*_AW ESP_ = 71.635 *P* < 0.001; IL-10: *t*_IIL ESP_ = 6.359, *P* = 0.001; *t*_AW ESP_ = 81.496 *P* < 0.001). Furthermore, compared with the IIL or AW ESP alone group, inhibition of RAW264.7 cell autophagy with 3BDO further elevated transcriptional levels of pro-inflammatory cytokines TNF-α and IL-1β (TNF-α: *t*_IIL ESP_ = -3.388, *P* = 0.019; *t*_AW ESP_ = -12.252, *P* < 0.001; IL-1β: *t*_IIL ESP_ = -3.671, *P* = 0.01; *t*_AW ESP_ = -4.355, *P* = 0.005), and 3BDO reduced transcription level of an*t*i-inflamma*t*ory cytokines TGF-β (*t*_IIL ESP_ = 5.627, *P* = 0.002; *t*_AW ESP_ = 68.096, *P* < 0.001), but 3BDO had no significant effec*t* on *t*he IL-10 transcription level (Fig 8A).

ELISA results revealed that IIL and AW ESP significantly increased the secretion of pro-inflammatory cytokines TNF-α and IL-1β in RAW264.7 cells (TNF-α: *t*_IIL ESP_ = 5.598, *P* = 0.005; *t*_AW ESP_ = 13.307, *P* < 0.001; IL-1β: *t*_IIL ESP_ = 26.627, *P* < 0.001; *t*_AW ESP_ = 19.218, *P* < 0.001); IIL and AW ESP also elevated obviously production of anti-inflammatory cytokines TGF-β and IL-10 (TGF-β: *t*_IIL ESP_ = 13.596, *P* < 0.001; *t*_AW ESP_ = 36.69, *P* < 0.001; IL-10: *t*_IIL ESP_ = 5.91, *P* = 0.004; *t*_AW ESP_ = 6.727, *P* = 0.003). Moreover, compared with the only IIL or AW ESP alone group, inhibition of RAW264.7 cell autophagy wi*t*h 3BDO further increased the secretion of pro-inflammatory cytokines TNF-α and IL-1β (TNF-α: *t*_IIL ESP_ = -4.269, *P* = 0.013; *t*_AW ESP_ = -4.66, *P* = 0.021; IL-1β: *t*_IIL ESP_ = -46.852, *P* < 0.001; *t*_AW ESP_ = -15.53, *P* < 0.001), but 3BDO reduced *t*he TGF-β secre*t*ion (*t*_IIL ESP_ = 8.695, *P* = 0.001; *t*_AW ESP_ = 31.505, *P* < 0.001), and had no significan*t* effect on the IL-10 secretion (Fig 8B).

These findings demonstrated that both IIL and AW ESP induced an inflammatory response in RAW264.7 cells, as characterized by obvious up-regulation of pro-inflammatory and anti-inflammatory cytokines. Moreover, autophagy inhibitor 3BDO further increased pro-inflammatory cytokines (TNF-α and IL-1β), but reduced anti-inflammatory cytokine TGF-β, suggesting that IIL and AW ESP-induced autophagy relieved RAW264.7 cell inflammation resulted from IIL and AW ESP.

### Autophagy alleviated Caco-2 monolayer disruption in Caco-2/RAW264.7 co-culture system

**Fig 9:**
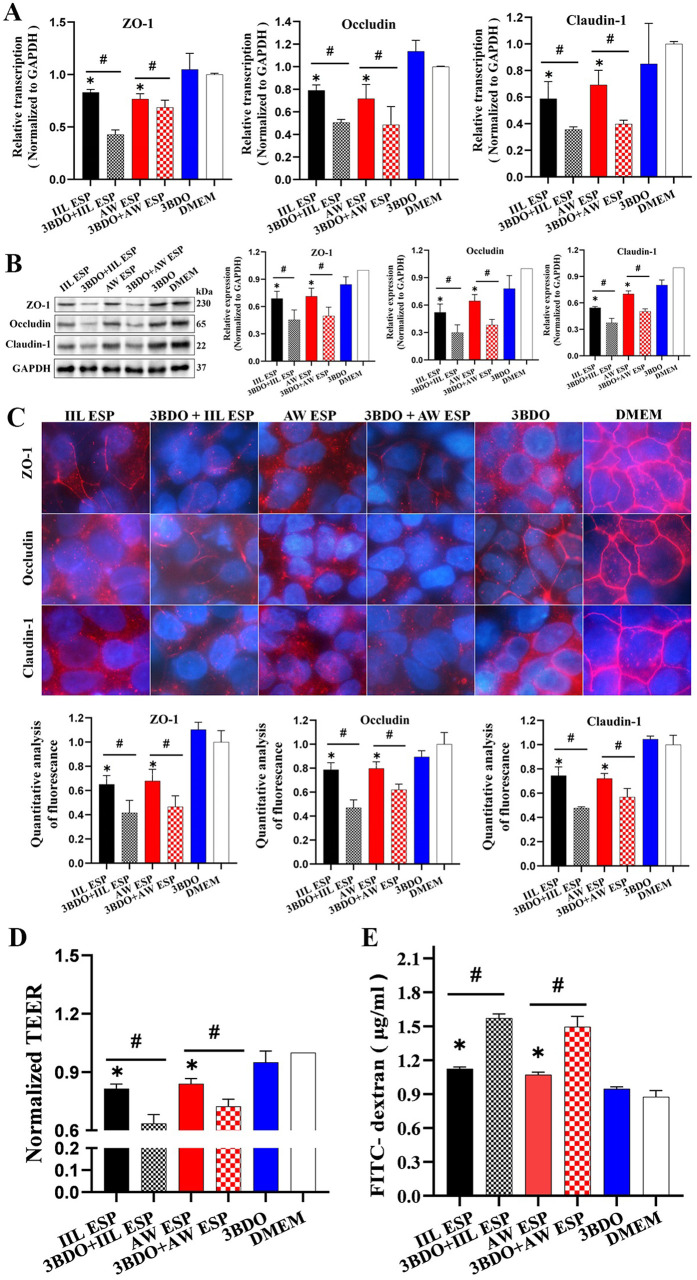
Autophagy protects IIL and AW ESP-induced the disruption of Caco-2 monolayer integrity in Caco-2/RAW264.7 co-culture model. **A:** qPCR analysis of transcription levels of TJs (ZO-1, Occludin and Claudin-1) in Caco-2 monolayer treated with IIL and AW ESP and 3BDO. **B:** Western blotting of expression level of TJs (ZO-1, Occludin and Claudin-1) in Caco-2 monolayer treated with IIL and AW ESP and 3BDO. **C:** IIFT analysis of expression level of TJs (ZO-1, Occludin and Claudin-1) in Caco-2 monolayer treated with IIL and AW ESP and 3BDO. **D:** TEER of Caco-2 monolayer treated with IIL and AW ESP and 3BDO. **E:** Paracellular permeability of Caco-2 monolayer by using FD-4 assessment after treatment IIL and AW ESP and 3BDO. Scale bars: 10 μm. Data are presented as mean ± SD of three independent assays. *Compared with the DMEM group, *P* < 0.05; ^#^compared with the only IIL ESP or AW ESP group, *P* < 0.05.

Total mRNA was extracted from Caco-2 cells. After reverse transcription into cDNA, the transcriptional levels of TJs (ZO-1, Occludin and Claudin-1) were assessed by qPCR. After treatment of RAW264.7 cells with IIL ESP or AW ESP, the transcriptional levels of ZO-1, Occludin and Claudin-1 in co-cultured Caco-2 cells were significantly reduced (ZO-1: *t*_IIL ESP_ = -13.867, *P* < 0.0001; *t*_AW ESP_ = -11.179, *P* < 0.0001; Occludin: *t*_IIL ESP_ = -10.522, *P* < 0.0001; *t*_AW ESP_ = -5.546, *P* = 0.003; Claudin-1: *t*_IIL ESP_ = -7.756, *P* < 0.0001; *t*_AW ESP_ = -6.836, *P* = 0.001). Furthermore, autophagy inhibition of both Caco-2 and RAW264.7 cells with autophagy inhibitor 3BDO further reduced transcriptional levels of ZO-1, Occludin and Claudin-1 in Caco-2 monolayers (ZO-1: *t*_IIL ESP_ = 19.155, *P* < 0.0001; *t*_AW ESP_ = 2.311, *P* = 0.043; Occludin: *t*_IIL ESP_ = 12.518, *P* < 0.0001; *t*_AW ESP_ = 2.785, *P* = 0.019; Claudin-1: *t*_IIL ESP_ = 4.346, *P* = 0.007; *t*_AW ESP_ = 6.412, *P* = 0.001) (Fig 9A).

Western blotting showed that when RAW264.7 cells in lower chamber were treated with IIL ESP or AW ESP, expression levels of ZO-1, Occludin and Claudin-1 of Caco-2 cells in upper chamber were significantly decreased (ZO-1: *t*_IIL ESP_ = -6.783, *P* = 0.002; *t*_AW ESP_ = -5.612, *P* = 0.03; Occludin: *t*_IIL ESP_ = -9.138, *P* = 0.001; *t*_AW ESP_ = -8.925, *P* = 0.012; Claudin-1: *t*_IIL ESP_ = -60.146, *P* < 0.0001; *t*_AW ESP_ = -15.234, *P* = 0.004). Autophagy inhibition of both Caco-2 and RAW264.7 cells by autophagy inhibitor 3BDO further decreased expression levels of ZO-1, Occludin and Claudin-1 in Caco-2 monolayers (ZO-1: *t*_IIL ESP_ = 3.031, *P* = 0.039; *t*_AW ESP_ = 2.848, *P* = 0.047; Occludin: *t*_IIL ESP_ = 3.079, *P* = 0.037; *t*_AW ESP_ = 4.933, *P* = 0.008; Claudin-1:*t*_IIL ESP_ = 5.747, *P* = 0.005; *t*_AW ESP_ = 7.775, *P* = 0.001) (Fig 9B).

The membrane of Trans-well chamber was cut off and used as a cell slide for IIFT to assess the expression levels of TJs on the surface of Caco-2 cells. Treatment of RAW264.7 cells with IIL ESP or AW ESP notably reduced expression levels of ZO-1, Occludin and Claudin-1 in the surface of co-cultured Caco-2 cells (ZO-1: *t*_IIL ESP_ = -5.073, *P* = 0.009; *t*_AW ESP_ = -4.124, *P* = 0.015; Occludin: *t*_IIL ESP_ = -3.251, *P* = 0.042; *t*_AW ESP_ = -3.012, *P* = 0.039; Claudin-1: *t*_IIL ESP_ = -4.211, *P* = 0.014; *t*_AW ESP_ = -5.529, *P* = 0.005). When autophagy of both Caco-2 and RAW264.7 cells was suppressed by the inhibitor 3BDO, the expression levels of TJs on Caco-2 monolayers were further decreased (ZO-1: *t*_IIL ESP_ = -4.124, *P* = 0.015; *t*_AW ESP_ = 2.812, *P* = 0.048; Occludin: *t*_IIL ESP_ = -3.121, *P* = 0.035; *t*_AW ESP_ = 4.315, *P* = 0.013; Claudin-1: *t*_IIL ESP_ = -5.529, *P* = 0.005; *t*_AW ESP_ = 3.267, *P* = 0.031) (Fig 9C).

These results indicated that autophagy inhibition of both Caco-2 and RAW264.7 cells by autophagy inhibitor 3BDO further decreased the transcription and expression of the TJs in Caco-2 monolayers, suggested that IIL and AW ESP-induced autophagy of Caco-2 and RAW264.7 cells alleviated Caco-2 monolayer integrity damage caused by IIL and AW ESP.

When RAW264.7 cells were incubated with IIL and AW ESP and inflammatory response occurred, the TEER of Caco-2 cell monolayers was significantly decreased (*t*_IIL ESP_ = -13.51, *P* = 0.005; *t*_AW ESP_ = -10.563, *P* = 0.009). Treatment of RAW264.7 cells with IIL and AW ESP also increased the paracellular permeability of Caco-2 monolayers (*t*_IIL ESP_ = 5.134, *P* = 0.002; *t*_AW ESP_ = 4.693, *P* = 0.004), suggesting that inflammatory response of RAW264.7 cells elicited by IIL and AW ESP indirectly increased paracellular permeability and disrupted integrity of Caco-2 monolayer. Moreover, when autophagy inhibitor 3BDO was used, the TEER was further decreased (*t*_IIL ESP_ = -5.976, *P* = 0.004; *t*_AW ESP_ = -4.531, *P* = 0.011) (Fig 9D), and paracellular permeability was further enlarged (*t*_IIL ESP_ = -27.256, *P* < 0.0001; *t*_AW ESP_ = -10.862, *P* < 0.0001) (Fig 9E).

These results indicated that in Caco-2/RAW264.7 co-culture model, inflammatory responses of RAW264.7 cells induced by IIL and AW ESP led to the TJs expression level decrease in Caco-2 cells, thereby compromising the Caco-2 cell monolayer integrity and barrier function. Conversely, autophagy of Caco-2 and RAW264.7 cell induced by IIL and AW ESP partially alleviated the damage of Caco-2 monolayer integrity, thereby contributing to the maintenance of the monolayer integrity.

## Discussion

Autophagy is an essential metabolic process for assisting in the degradation of dysfunctional cell components or exogenous pathogens through the lysosomal system to attain cellular homeostasis [48]. The mammalian target of rapamycin (mTOR) kinase is a key protein molecule for inducing autophagy. The Akt and MAPK signaling pathway suppress autophagy via activating the mTOR pathway. On the contrary, the AMPK pathway facilitates autophagy by negatively regulating mTOR pathways. Autophagy acts a primary role through cleaning misfolded or aggregated molecules and removing damaged organelles [49]. Autophagy also destroys the invaded exogenous pathogens (virus, bacterium and parasite). Autophagy participated in eliminating *Toxoplasma gondii* tachyzoites by stripping the parasite plasma membrane [50]. Moreover, host cell autophagy induced by *T. gondii* and *Plasmodium* is beneficial to their growth and development [51,52].

The autophagy role in some intestinal and intracellular protozoon infections has been reported. *Cryptosporidium parvum* infection induced autophagy in Caco-2 cells by inhibiting mTOR. After silencing ATG7 (a key autophagy protein) with siRNA, *C. parvum*-induced reduction of TJs proteins (Occludin, Claudin-4 and E-cad) was partially blocked [53]. *Toxoplasma gondii* infection induced autophagy in host intestinal Paneth cells, and this autophagic response is crucial for protecting against *T. gondii*-induced intestinal damage [54]. Previous studies also showed that *T. spiralis* infection induces autophagy of murine IECs. From the beginning of 3 days post-infection, autophagy was observed in the duodenal, jejunal, and ileal tissues of *T. spiralis*-infected mice, as demonstrated by an increase of autophagosomes and autolysosomes, elevated expression of autophagy marker proteins Beclin-1 and LC3B-II/LC3B-I, and a reduction of autophagy substrate p62 levels [8]. *T. spiralis* infection induced autophagy of host skeletal muscle cells, but the ML ESP suppressed the autophagy of myoblasts *in vitro* [55]. However, the autophagy roles at the process of *T. spiralis* invasion and colonization are not clear. The IIL and AW are two crucial enteral invasive stages in *T. spiralis* infection [5,56]. *T. spiralis* IIL ESP bound with RACK1 receptor in Caco-2 cells and increased the RACK1 expression levels [28]. Over expression of RACK1 significantly increased intracellular Ca^2+^ concentration; conversely, weakening RACK1 expression via RNA interference (RNAi) obviously reduced Ca^2+^ release and decreased intracellular Ca^2+^ concentration [57]. In this study, IIL and AW ESP were used to testify whether they induce Caco-2 cell autophagy and to investigate the relationship between ESP-induced gut epithelial autophagy and intestinal epithelial barrier integrity.

Eukaryotes regulate their metabolism according to the availability of nutrients in the environment; a key player in this process is the AMP-activated protein kinase (AMPK). In higher eukaryotes, AMPK senses the intracellular available energy status by directly binding adenine nucleotides. AMPK has two major upstream regulatory kinases, and they are serine/threonine kinases. The first is liver kinase B1 (LKB1), and the second is Ca^2+^/calmodulin-dependent kinase IIβ (CaMKKβ). LKB1 and CaMKKβ activate AMPK in response to energy stress (signaled by elevated AMP levels) and increased intracellular Ca^2+^ levels, respectively [58]. Overexpression of CaMKK increases AMPK activity, while inhibiting CaMKKβ decreases AMPK activity [59]. Therefore, an increase in intracellular Ca^2+^ levels may activate AMPK. AMPK is an upstream molecule in the mTOR pathway. Once being activated, AMPK inhibits mTOR complex 1 (mTORC1) activity on the one hand and positively regulates ULK1 activity through phosphorylation on the other hand, promoting the assembly of autophagy initiation complexes and the occurrence of autophagy [60].

In this study, our results showed that IIL and AW ESP increased the RACK1 expression in both Caco-2 and RAW264.7 cells, and simultaneously elevated intracellular Ca^2+^ concentration and activated the AMPK pathway. When the RACK1 expression in Caco-2 and RAW264.7 cells was inhibited by the RACK1 inhibitor HO, the Ca^2+^ concentration was decreased and the activation of AMPK pathway was also suppressed, indicating that IIL and AW ESP increased RACK1 expression to elevate Ca^2+^ concentration in Caco-2 and RAW264.7 cells and activated AMPK. Additionally, IIL and AW ESP induced inhibition of p-mTOR in Caco-2 and RAW264.7 cells, accompanied by increased expression of autophagic marker protein Beclin-1 and reduced levels of the autophagic substrate p-62, suggesting that both IIL and AW ESP promoted autophagy of Caco-2 and RAW264.7 cells. When AMPK activation was inhibited by the AMPK inhibitor DD, all the IIL and AW ESP-induced the p-mTOR inhibition, the increased Beclin-1expression and the reduced levels of autophagic substrate p-62 were also reversed. These results indicated that IIL and AW ESP promoted autophagy of Caco-2 and RAW264.7 cells by increasing RACK1 expression, elevating intracellular Ca^2+^ concentration, and activating the AMPK/mTOR signaling pathway.

Autophagy protects the structural and functional integrity of intestinal epithelial barrier. Autophagy enhances the intestinal epithelium barrier function by promoting the expression of TJs proteins (ZO-1 and Occludin) and their distribution to the cell surface [15,16]. Autophagy also reduced the degradation and facilitated the membrane localization of Occludin to strengthen enteral epithelium TJs barrier function [9].

Autophagy prevents cytokine-mediated disruption of intestinal epithelial barrier by regulating cytokine expression. The regulatory role of autophagy on intestinal inflammation has been widely confirmed [17,18]. Cytokines also have a significant regulatory effect on intestinal epithelial TJs proteins. TNF-α, a key pro-inflammatory cytokine, reduced the expression levels of ZO-1 and Occludin in Caco-2 cells, while enhanced the Caco-2 monolayer permeability [61,62]. IL-1β induced the increase of TJs permeability in Caco-2 cells, which is associated with reduced expression of ZO-1 and Claudin-1, as well as decreased surface localization of Occludin [63]. The cytokine effect on intestinal epithelial integrity has also been confirmed in mice [64]. TGF-β enhanced the barrier function of human colonic epithelial T84 and HT-29 cell monolayers and protected against barrier disruption induced by enterohemorrhagic *Escherichia coli* O157:H7 (EHEC) in T84 cell monolayers, which is achieved by maintaining the protein expression levels of ZO-1 and Occludin [65]. The IL-10 ameliorated the TNF-α-induced barrier damage in Caco-2 cell monolayers [66]. Our results showed that when Caco-2 cell autophagy was inhibited with autophagy inhibitor 3BDO, the IIL and AW ESP-reduced expression levels of TJs (ZO-1, Occludin and Claudin-1) in Caco-2 monolayers were further decreased. Additionally, the TEER of Caco-2 monolayer was further decreased, and paracellular permeability was further enlarged. These findings indicated that IIL and AW ESP disrupted the integrity and barrier function of Caco-2 monolayer, and autophagy inhibitor further aggravated the disruption of the monolayer integrity, suggesting that IIL and AW ESP-induced Caco-2 cell autophagy also partially protect the Caco-2 monolayer integrity. Based on this finding, it is speculated that the TsESP’s destructive effects during *T. spiralis* infection might be attenuated through gut epithelial autophagy, thereby maintaining intestinal epithelial cellular homeostasis and intestinal worm survival in intramulticellular niche of gut epithelium [1].

In *T. spiralis* infection, immune cells are more critical than epithelial cells in producing cytokines in response to the parasite itself and its ESP. Macrophages are the primary cytokine-producing cells [67]. Therefore, in this study, the transcriptional and secretory levels of cytokines in both Caco-2 and RAW264.7 cells were ascertained after they were treated by IIL and AW ESP. The results confirmed that IIL and AW ESP increased the expression levels of pro-inflammatory (TNF-α and IL-1β) and anti-inflammatory cytokines (TGF-β and IL-10) in both Caco-2 and RAW264.7 cells. After autophagy of Caco-2 and RAW264.7 cells was suppressed by autophagy inhibitor 3BDO, expression levels of pro-inflammatory cytokines (TNF-α and IL-1β) were further elevated, while anti-inflammatory TGF-β was decreased. The results indicated that IIL and AW ESP-induced autophagy inhibited the secretion of pro-inflammatory cytokines and promotes the secretion of anti-inflammatory cytokines in Caco-2 and RAW264.7 cells, which is beneficial to repair the damaged gut epithelium. However, it is regrettable that the effect of autophagy inhibition on IL-10 secretory levels in Caco-2 and RAW264.7 cells was not detected in this study.

To further validate the cytokine effect on Caco-2 monolayer barrier under IIL and AW ESP stimulation and to investigate the role of autophagy in this process, a Caco-2/RAW264.7 co-culture model was used in this study. The results showed that when RAW264.7 macrophages were stimulated with IIL and AW ESP, transcriptional and expression levels of TJs (ZO-1, Occludin and Claudin-1) in Caco-2 cell monolayer were significantly reduced, while TEER was obviously declined, and permeability was distinctly increased. When autophagy of Caco-2/RAW264.7 in co-culture system was inhibited by 3BDO, the protective effect of autophagy on TJs in Caco-2 monolayer was weakened, and its regulatory effect on cytokine production of Caco-2 cells and RAW264.7 cells was diminished, leading to more severe destruction of Caco-2 monolayer integrity. The results further confirm that cytokines produced by RAW264.7 macrophages treated with IIL and AW ESP disrupted the integrity of co-cultured Caco-2 monolayer barrier. During the process, IIL and AW ESP-induced autophagy protected the integrity of Caco-2 monolayer barrier in co-culture system.

After the encapsulated *T. spiralis* ML are liberated from their capsules under digestion fluid, the ML are activated into the IIL by enteral contents and bile, the IIL penetrate enteral epithelium where the IIL establish their intramulticellular niche. This niche is composed of a row of columnar IECs intruded by the IIL. Surprisingly, the IIL penetration of host IECs does not lead to the damage and disintegration of host cells; instead, these mucosal epithelium cellular membranes are fused with each other to form a syncytium to accommodate the nematode larva. The worms lived in the intramulticellular niche evade the killing and clearance by host’s intestinal immune response. The IIL develop into adult worms within the niche of host’s intestinal columnar epithelium and produce newborn larvae [1]. Therefore, in *T. spiralis* life cycle, the worms and their ESP also have functions to limit the damage of enteral epithelium in addition to disrupting host’s intestinal epithelium to mediate larval invasion [34,68]. These mechanisms maintain the niche required by the worms, allowing them to colonize the host’s intestine and continue to develop, rather than being exposed to intestinal lumen and being killed or cleared by the immune response, which ensure the survival of both the host and parasite [69].

In conclusion, our results showed that the ESP from two *T. spiralis* intestinal stages (IIL and AW) induced autophagy of Caco-2 and RAW264.7 cells through up-regulating RACK1 expression, increasing intracellular Ca^2+^ concentration, and activating AMPK/mTOR signaling pathway. Although IIL and AW ESP disrupted Caco-2 monolayer integrity, autophagy induced by IIL and AW ESP also abolished and alleviated the ESP decreased-TJs expression levels in Caco-2 monolayer, reduced the ESP-induced secretion of pro-inflammatory cytokines (TNF-α and IL-1β), and enhanced ESP-upregulated production of anti-inflammatory cytokines (TGF-β). Therefore, under the *in vitro* conditions, TsESP-induced autophagy ultimately alleviated and limited the intestinal epithelial damage caused by TsESP itself. During *T. spiralis* infection, the TsESP might be beneficial to the development and survival of the IIL and adult worms in host intestinal mucosal epithelium through inducing gut epithelial autophagy and maintaining gut homeostasis. However, the relationship among intestinal epithelial autophagy, intestinal barrier damage and immune regulation in *T. spiralis* infection needs to be validated by using intestinal organoids or animal experiment in future research.

## Supporting information

S1 Fig Autophagy of Caco-2 cells incubated with 15 μg/ml IIL ESP for 0, 12, 24 and 36 h. IIL ESP significantly up-regulated the Beclin-1 expression level in Caco-2 cells at 12 h post incubation; meanwhile IIL ESP reduced p62 protein levels at 12–36 h post incubation. *Compared with pre-incubated (0 h) group, *P* < 0.05.
(TIF)
