# Supplementary figures and images for: Trichinella spiralis excretory-secretory proteins induced autophagy via activating AMPK/mTOR pathway and protected gut epithelial barrier
Source: PLoS Negl Trop Dis. 2025 Dec 22;19(12):e0013863. doi: 10.1371/journal.pntd.0013863 (PMC12818875; doi:10.1371/journal.pntd.0013863)

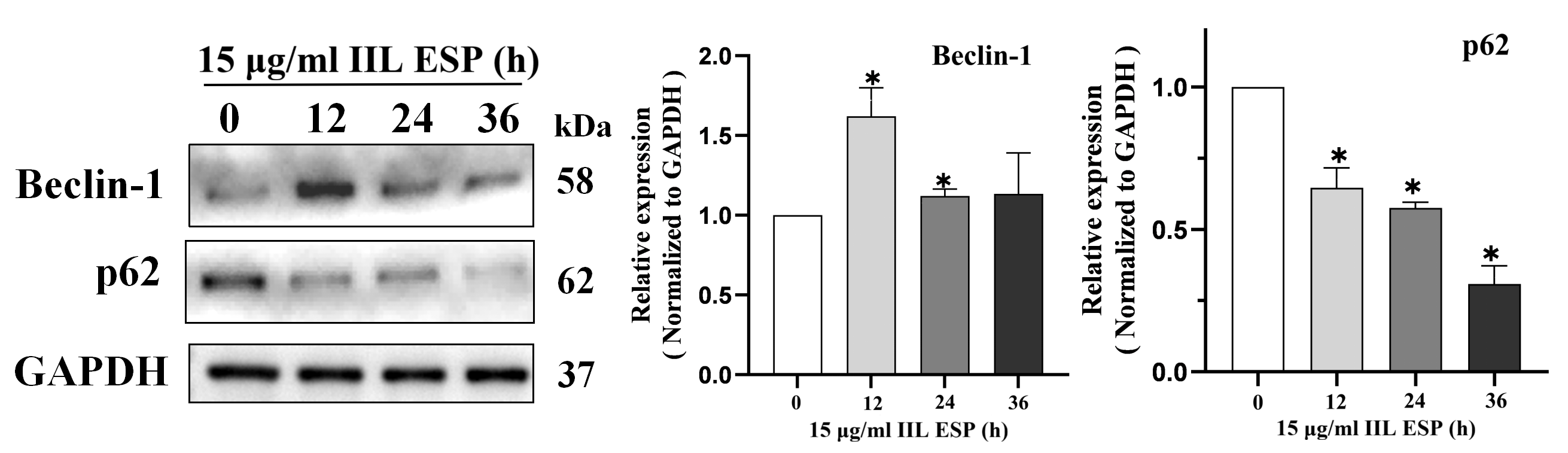

Supplement: S1 Fig — IIL ESP significantly up-regulated the Beclin-1 expression level in Caco-2 cells at 12 h post incubation; meanwhile IIL ESP reduced p62 protein levels at 12–36 h post incubation. *Compared with pre-incubated (0 h) group, P < 0.05. (TIF) [file pntd.0013863.s001.tif]
